# Evaluating the accuracy of protein design using native secondary sub-structures

**DOI:** 10.1186/s12859-016-1199-y

**Published:** 2016-09-05

**Authors:** Marziyeh Movahedi, Fatemeh Zare-Mirakabad, Seyed Shahriar Arab

**Affiliations:** 1 Department of Mathematics and Computer Science, Amirkabir University of Technology, Tehran, Iran; 2 Department of Biophysics, Faculty of Biological Sciences Tarbiat Modares University (TMU), Tehran, Iran

**Keywords:** Protein design, Protein structure prediction, Evolutionary information

## Abstract

**Background:**

According to structure-dependent function of proteins, two main challenging problems called Protein Structure Prediction (PSP) and Inverse Protein Folding (IPF) are investigated. In spite of IPF essential applications, it has not been investigated as much as PSP problem.

In fact, the ultimate goal of IPF problem or protein design is to create proteins with enhanced properties or even novel functions. One of the major computational challenges in protein design is its large sequence space, namely searching through all plausible sequences is impossible. Inasmuch as, protein secondary structure represents an appropriate primary scaffold of the protein conformation, undoubtedly studying the Protein Secondary Structure Inverse Folding (PSSIF) problem is a quantum leap forward in protein design, as it can reduce the search space.

In this paper, a novel genetic algorithm which uses native secondary sub-structures is proposed to solve PSSIF problem. In essence, evolutionary information can lead the algorithm to design appropriate amino acid sequences respective to the target secondary structures. Furthermore, they can be folded to tertiary structures almost similar to their reference 3D structures.

**Results:**

The proposed algorithm called *GA*PSSIF benefits from evolutionary information obtained by solved proteins in the PDB. Therefore, we construct a repository of protein secondary sub-structures to accelerate convergence of the algorithm.

The secondary structure of designed sequences by GAPSSIF is comparable with those obtained by Evolver and EvoDesign. Although we do not explicitly consider tertiary structure features through the algorithm, the structural similarity of native and designed sequences declares acceptable values.

**Conclusions:**

Using the evolutionary information of native structures can significantly improve the quality of designed sequences. In fact, the combination of this information and effective features such as solvent accessibility and torsion angles leads IPF problem to an efficient solution. GAPSSIF can be downloaded at http://bioinformatics.aut.ac.ir/GAPSSIF/.

**Electronic supplementary material:**

The online version of this article (doi:10.1186/s12859-016-1199-y) contains supplementary material, which is available to authorized users.

## Background

Proteins are building blocks of life, serving main roles in the body. Since the function of a protein is dependent on its structure, some experimental methods are applied for tertiary structure determination. Not only these methods are time-consuming and expensive but also they cannot build a proper atomic model for some proteins. Thus, computational methods have been known as favorable approaches for protein structure prediction (PSP) within the last two decades.

In PSP problem, an amino acid sequence is given as an input and the goal is to predict the best-adapted structure respective to its function. In this regard, another essential problem called protein design or inverse protein folding (IPF) [1–3] is defined to identify a sequence of amino acids whose tertiary structure corresponds to a given target structure. Indispensable applications of IPF in drug design, medicine and advanced disease treatment evoked scientists to develop methods for designing appropriate sequences. Unfortunately, because of IPF NP-Hardness [4], it is impossible to give an exact algorithm to solve this problem.

First attempts to solve this problem back to late 1980s which mainly focused on amino acid compositions of designed sequences [1]. In 1988 Ragan and Degrado had a somewhat successful design for a 4-helix bundle structure [5]. Later, Yue and Dill [2] developed a high simplified model, called Hydrophobic-polar, embedded in a cubic lattice. This model was developed according to the structural pattern in globular proteins where hydrophobic and polar residues, respectively form internal core and surface of the protein. Many attempts have been done to extend lattice-based methods such as approximation algorithms [6, 7].

In 1994, a multi-objective genetic algorithm was developed by Jones to solve IPF problem, in which the input of algorithm is a protein secondary structure [8]. However, improvements in proteomics including protein force fields [10,9, 11] and rotamer libraries [12] enabled scientists to solve this problem in the atomic level. In this era, several algorithms were developed to find the best sequences through the solution space using energy functions [13, 14]. Besides, they take into account the effects of amino acid conformations, commonly in the form of “rotamer libraries”. The essence of IPF solutions, up to 2012, was to find an amino acid sequence which folds to a low energy structure by means of assigning more hydrophobic residues or minimizing a protein energy function.

Due to the simplifications of folding driving forces by protein design models including discrete rotamer space and approximate energy functions, IPF problem was incapable to reach its holy grail. Until 2012, the point which had been ignored was the evolutionary information derived from protein databases. Recently, EvoDesign [3, 15] has been developed to take into account the evolutionary information in form of profile collections obtained by native structures of the PDB database. As it was mentioned in [3], several methods in the literature were developed to design specific proteins, but modern methods should be able to design sequences for any protein scaffold. Despite the abovementioned de novo protein design algorithms, Evolver [16, 17] has another point of view which evolves three different types of protein sequences for each input target structure using simulated annealing. The first one is the native sequence of input structure extracted from the PDB database. While, the second one is obtained by shuffling the native sequence and the last one is a random protein-like sequence.

Since IPF is the reverse procedure of protein folding, any suitable method to solve this problem should employ folding driving forces. As the folding initially involves the establishment of regular structures, in particular alpha helices and beta sheets, secondary sub-structures would be useful in solving PSSIF problem. Actually, these regular structures can make an appropriate scaffold of protein tertiary structure; furthermore, they can affect amino acid composition in primary structure through evolution process. In general, importance of PSSIF problem arises from the fact that secondary structure is one the most effective features in tertiary structure and function of proteins.

This paper involves native secondary sub-structures as evolutionary information to improve designing process. Thus, a novel genetic algorithm, named GAPSSIF, using these sub-structures is proposed to solve PSSIF problem. In other words, a precise protein repository is constructed by extracting all possible protein secondary sub-structures from PDB. In this algorithm, each individual takes advantage of a knowledge-based procedure using the sub-structure repository. In essence, evolutionary information can lead the algorithm to design appropriate amino acid sequences respective to the target secondary structures. Furthermore, they can be folded to tertiary structures almost similar to their reference 3D structures. GAPSSIF is compared with two well-known algorithms called EvoDesign and Evolver. The assessment of proposed algorithm on 89 non-redundant proteins confirms the strong performance in solving PSSIF. In addition, the predicted tertiary structures of designed sequences represent acceptable results.

## Method

In this section, a genetic algorithm [18] called GAPSSIF is presented to solve PSSIF problem. This algorithm makes use of evolutionary information through PDB secondary structure elements. Thus, prior to explain the proposed algorithm, constructing a repository of protein secondary sub-structure elements is described.

### Building up sub-structure repository

A collection of 102101 proteins was derived from the PDB secondary structures file generated using DSSP [19]. It contains all existing amino acid sequences as well their secondary structures. Since PDB is highly redundant, proteins with more than 90 % sequence identity were omitted to eschew bias of designed sequences to a specified group of proteins. Afterwards, corresponding amino acid fragments were extracted for all helices (H), beta sheets (E) and all other kinds of secondary structure elements (C).

Eventually, by fetching amino acid fragments for each distinct sub-structure, an Amino Acid Fragment Repository (AFR) which highly increases the precision of proposed algorithm, is formed. This repository comprises three main clusters including helix, beta sheet and coil. Each sub-cluster contains non-identical amino acid fragments with a specified length. For example, a sub-cluster named *“H*11*SC”* includes some fragments with 11 amino acids whose secondary structures are Helix. In essence, we represent a sub-cluster with *ekSC* where *e* ∈ {*E, H, C*} and *k* assigns the length of sub-structure *e*. There are totally 306 sub-clusters in the repository, 38 for Beta strands, 141 for Helices and 127 for Coils. Clearly, some lengths do not exist among PDB peptides.

### The proposed algorithm to solve PSSIF problem

In this subsection, we aim to describe the steps of GAPSSIF for solving PSSIF problem to design appropriate amino acid sequences folded to target secondary structures. The following mathematical definition outlines PSSIF problem:

$$ \begin{array}{l}\boldsymbol{Input}:SS=s{s}_1\dots s{s}_l,s{s}_i\in \varGamma =\left\{E,H,C\right\},\\ {}\boldsymbol{Output}:\ S = {s}_1\dots {s}_l,{s}_i\in {\displaystyle \sum =\Big\{20\kern0.5em  standard}\kern0.5em  amino\kern0.5em  acids\Big\}.\end{array} $$

Algorithm 1 depicts an overview of the proposed method. In the first step of GAPSSIF, the input secondary structure is split into a set of sub-structures, elements, as described below:

1 $$ su{b}_{ss}=\left\{<\sigma, k,e{>}_j\Big|e\in \varGamma \& s{s}_{\sigma -1},s{s}_{\sigma +\mathrm{k}}\ne e\&\sigma, k=1,\dots, l\right\}, $$

where *σ*, *k* and *e* indicate respectively, the start position, length and type of *jth* element in the *l*-length target structure.

#### Creating initial population

This subsection describes the second step of GAPSSIF (algorithm 1) to make an appropriate initial population where each individual of the population is a 2-tuple < *S*_*i*_*, Fit*_*i*_ > whose *S*_*i*_ is made up of 20 standard amino acids as follows:

$$ {S}_i={F}_1\dots {F}_{\left|su{b}_{ss}\right|}, $$

where *F*_*j*_ shows the corresponding amino acid fragment for *jth* element, <*σ, k, e* > *j*, of *sub*_*ss*_ and it is built up as follows:

2 $$ {F}_j=\Big\{\begin{array}{l} ExactFragment\left(k,e\right)\kern1em \\ {} Neighboring\left(k,e\right)\kern1em \\ {} ChoFaGenerator\left(k,e\right)\kern1em \end{array}\begin{array}{l} ekSC\in AFR\wedge r\in \left[0,0.7\right),\kern1em \\ {}\left( ekSC\in AFR\wedge r\in \right[0.7,0.95\left)\right)\vee ( ekSC\notin AFR\wedge r\in \left[0,0.9\right))\\ {} Otherwise,\end{array}\kern1em \operatorname{}, $$

where *ekSC* represents a sub-cluster in AFR (see “Building up sub-structure repository”) and *r* ∈ [0,1) is a random value. In equation (2), *ExactFragment* procedure is applied if the intended sub-cluster exists and the random value *r* ∈ [0,0.7). This procedure randomly fetches an amino acid fragment from *ekSC*. In contrast to the first case of equation (2), the second case occurs if the intended *ekSC* exists and the random value *r* ∈ [0.7,0.95) or intended *ekSC* does not exist in AFR but the random value *r* ∈ [0,0.9). In general, *Neighboring* procedure is employed to edit a shorter or longer element of the repository, see algorithm 2. Steps 1, 2 and 3 of this algorithm find *ek′SC* sub-cluster with the lowest difference from *k*. Afterwards, in step 4, *ExactFragment* procedure is used to fetch a fragment from *ek′SC* sub-cluster and then, step 5 modifies the fetched fragment to length *k*.

In case 3 of equation (2), if none of the two first cases is satisfied, the required fragment is generated by *ChoFaGenerator* procedure using *ChoFaWeight* (CFW) function. According to Chou-Fasman [20] analysis on secondary structure dependent propensities, amino acids have various tendencies to participate in each secondary sub-structure or element. Therefore, CFW function applies roulette wheel selection through 20 standard amino acids in order to select an appropriate residue.

Accordingly, using evolutionary information in creating amino acid sequences results in a better starting point to search through the sequence space and substantially accelerates convergence of the algorithm. In order to calculate the fitness value of generated amino acid sequence (*S*_*i*_), two main steps should be taken. At first, the secondary structure of *S*_*i*_ called *PSS*_*i*_ = *pss*_i1_…*pss*_*il*_ is predicted by Reprof [21]. Secondly, the similarity is computed between the predicted secondary structure, *PSS*_*i*_, and target structure, SS, as described below:

$$ \begin{array}{l}Fi{t}_i={\displaystyle \sum_{j=1}^l{\chi}_{s{s}_j}}\left( ps{s}_{ij}\right),\\ {}{\chi}_h\left({h}^{\prime}\right)=\Big\{\kern1em \begin{array}{c}1,\kern2em \\ {}\kern1em 0,\kern1em \end{array}\kern1em \operatorname{}\kern1em \begin{array}{c}h={h}^{\prime },\kern1em \\ {}\kern1em h\ne {h}^{\prime }.\kern1em \end{array}\end{array} $$

Eventually, |*sub*_*ss*_| individuals are generated using aforementioned processes to construct an initial population.

#### Enriching amino acid individuals

In the third step of proposed algorithm (algorithm 1), each individual is enriched using Gibbs Sampling algorithm. This method employs AFR Mutation (AFRM) operation iteratively to fortify the individuals using evolutionary information in AFR.

In the first step of Gibbs Sampling method, AFRM operation takes individual *S*_*i*_ and its predicted secondary structure, *PSS*_*i*_, to generate *P'SS*_*i*_ which specifies incorrectly predicted positions of *PSS*_*i*_ as follows:

$$ {P}^{\prime }S{S}_i={p}^{\prime }s{s}_{\mathrm{i}1}\dots {p}^{\prime }s{s}_{il}, $$

where

$$ {p}^{\prime }s{s}_{\mathrm{ij}}=\Big\{\kern1em \begin{array}{cc}-\kern1em & \kern1em {\chi}_{s{s}_j}\left( ps{s}_{\mathrm{ij}}\right)=1,\kern1em \\ {}\kern1em  ps{s}_{\mathrm{ij}}\kern1em & \kern3.5em  else.\end{array}\kern1em \operatorname{} $$

Then, pattern *P'SS*_*i*_ is split into a set of secondary sub-structures as described below:

$$ su{b}_{P^{\prime }S{S}_i}=\left\{<\sigma, k,e{>}_j\Big|e\in \varGamma \&s{s}_{\sigma -1},s{s}_{\sigma +1}\ne e\&\sigma, k=1,\dots, l\right\}. $$

In the following, for each element in set $su{b}_{P^{\prime }S{S}_i}$, a fragment is built according to the equation (2). At last, these fragments are located on the corresponding fragments in sequence *S*_*i*_ to generate a new sequence called *newS*_*i*_. Then, the fitness value of *newS*_*i*_ is computed and named *newFit*_*i*_.

In the second step, Gibbs Sampling method replaces sequence *S*_*i*_ with designed sequence *newS*_*i*_ when *newFit*_*i*_ is greater than *Fit*_*i*_. The first and the second steps of Gibbs Sampling are conducted |*sub*_*ss*_| times.

#### Constructing Hit Map Repository

In step 4 of GAPSSIF (algorithm 1), Hit Map Repository (HMR) is constructed to contain all correctly designed subsequences whose structures are identical to the corresponding elements of target structure. Each identical element is represented as follows:

$$ T=\left\{< key,s>\right\}, $$

where “key” shows the structure of subsequence *s* in structure *PSS*. For instance, <(*B*4*, H*3*, C*2)*, s* > indicates that there is a subsequence *s* in the designed sequence whose structure consecutively contains a beta-sheet, alpha helix and coil respectively with lengths four, three and two.

In fact, hit map repository is the result of complementary collaboration between AFR and secondary prediction algorithm. It means that HMR comprises those fragments which are accepted by both evolution process and the secondary structure predictor. In other words, HMR consists of multi-structural fragments which are simulated during the algorithm using both prediction algorithm and AFR.

#### Mutation operations

GAPSSIF employs two mutation operations to mutate individuals in step 5. Each individual is mutated randomly using AFRM (see “Enriching amino acid individuals”) or HMR Mutation (HMRM) operations. The first operation, AFRM, was described as a part of Gibbs Sampling method in “Enriching amino acid individuals”. The second one, HMRM, employs hit map repository to mutate a designed sequence, *S*_*i*_, to generate an offspring named *newS*_*i*_ as described in algorithm 3. HMRM operation tries to find a proper multi-structural fragment from HMR to locate in *S*_*i*_. Finally, the fitness values of mutated individuals are computed and added as new individuals to the population *P*.

Eventually, in step 6 of algorithm 1, the extended population is sorted in descending order based on the fitness values of individuals. Afterwards, in step 7, extra individuals are removed from the population till the new generation reaches the size of initial population. GAPSSIF is repeated until a solution with identical secondary structure to the target is found or goes on for 50 iterations. According to the length of the largest sub-structure in the benchmark, the maximum number of iterations is set to 50.

## Results and discussion

GAPSSIF was implemented using Perl and all calculations were done on an Intel core i7-3770 processor (8M Cache, 3.40GHz) with 16GB RAM in 64bit Ubuntu Linux.

In this section, the quality of 2D and 3D structures of designed sequences by GAPSSIF are evaluated. Thus, a set of 89 non-redundant proteins is used with different lengths vary from 52 to 196 amino acids [3]. According to Structural Classification of Proteins (SCOP) [22], the selected dataset includes 9 alpha (α), 18 beta (β), 26 alpha + beta (α + β), 11 alpha/beta (α/β) and 2 small proteins.

### GAPSSIF evaluation on a non-redundant dataset

This subsection presents GAPSSIF evaluation on 89 proteins. With reference to heuristic nature of GAPSSIF, it was executed ten times for each PDB ID of Additional file 1: Table S1. It should be noted that the best designed sequence in these 10 executions is the one with higher accuracy (Q3) and less iterations.

An investigation over Additional file 1: Table S1 shows significant success of GAPSSIF in designing amino acid sequences for the target secondary structures. *Column (a)*, PSD-Q3, represents the percentage of similarity between target and predicted secondary structure of designed sequence [23]. In addition, *column (b),* SOV, illustrates the segment overlap score which is based on the average overlap between the reference and designed segments [23]. As it is shown by Q3 and SOV, the proposed algorithm successfully designed appropriate sequences for 89 proteins with different lengths and folding classes. In 88 samples, resultant sequences have identical secondary structures to the target structure. Even in 1NXM, there is just one residue with non-identical secondary structure. Furthermore, the value of *column (c)* which specifies the iteration number of the algorithm demonstrates the high convergence of GAPSSIF. Even in 1NXM, the best possible sequence was designed just through 7 iterations and it did not change till termination condition. Meanwhile, *column (d)* shows the execution time for making and enriching initial population using AFR. Moreover, *column (e)* indicates the execution time to search through the solution space using genetic algorithm operations. Thus, *column (f)* refers to the total time of proposed algorithm given by the summation of *columns (d)* and *(e)* plus one second for loading AFR. Generally, the process of making initial population is in the order of *O*(*l*^2^) and the time complexity of iteration is *O*(*l*^2^) where *l* shows the length of target secondary structure. Moreover, the space complexity of this algorithm is also in the order of *O*(*l*^2^) to save hit map repository and individuals in each generation.

The values of *column (g)* illustrate normalized difference of amino acid compositions between designed sequence *S* = *s*_1_…*s*_*l*_ and reference sequence *R* = *r*_1_…*r*_*l*_ as follows:

$$ NDC=\frac{1}{20}{\displaystyle \sum_{j\in \varSigma}\left|{\displaystyle {\sum}_{i=1}^l{\chi}_j\left({r}_i\right)}-{\displaystyle {\sum}_{i=1}^l{\chi}_j\left({s}_i\right)}\right|}. $$

The zero value of *NDC* shows that amino acids distribution in designed sequences is typical of their references. However, the rationale of having NDC value greater than zero is the one behind PAM or BLOSUM substitution matrices, namely some amino acids are mutable to one another. In this regard, the low sequence and fragment identities in *columns (h) and (i)* not only mitigate the conjecture of using the reference sequence from AFR in designing sequence for the corresponding structure, but also show high diversity of designed sequences. As it is marked by “#” in Additional file 1: Table S1, fortunately just five proteins have non-zero fragment identity. In fact, high sequence identity cannot validate the quality of designed sequences alone, since PDB database has been completed from structural perspective not amino acid sequences. As we know many amino acid sequences can be folded to one protein conformation. In addition, this high diversity could be more useful for practical applications such as biological or chemical purposes. Meanwhile, amino acid composition variance in *column (j)* demonstrates that the designed protein amino acid compositions are typical of the input scaffold folding class [8]. The number of successful hits in *column (k)* emphasizes that there is an appropriate designed sequence in almost all ten independent runs of the proposed algorithm. In *column (l*), the average value of 99.59 for all 890 designed sequences (for each of 89 proteins, ten sequences are designed by GAPSSIF) confirms remarkable achievement of GAPSSIF in solving PSSIF problem. The success of proposed algorithm is due largely to the evolutionary information and the simulation of multi-structural fragments. *Column (m)* indicates that although in some executions the predicted secondary structure of designed sequences is not identical to the target structure, the algorithm is able to design sequences with few incorrect residues. It is clear that the zero values in this column clarify a successful design in all ten executions. In order to better represent the simultaneous effect of AFR and HMR, the predicted secondary structure accuracy of reference sequences is shown in the last column of Additional file 1: Table S1. In fact, the limitations imposed by prediction algorithms intentionally are used to enhance the performance of GAPSSIF. To be more specific, for each secondary structure segment we have two possible repositories, the first one is authorized by nature-evolved sequences and the second includes common fragments which are acceptable by both nature and predictor. In fact, GAPSSIF uses a prediction algorithm not only to evaluate individuals as a fitness assessor but also to play an effective role in constructing amino acid sequences. Although, the prediction accuracy of a reference sequence is restricted (see *column n*) even in the best secondary structure predictors, threat can turn into opportunity by the complementary collaboration of evolutionary and simulated data.

It should be mentioned that in order to cross-validate evaluation procedure, PDB IDs in Additional file 1: Table S1 marked with “*” were omitted while creating AFR. Eliminating 1Y25, 1V5I, 2WLV, 2ERb and 3FIL does not affect GAPSSIF good performance. Moreover, despite the existence of 1NXM in AFR, it does not have any exact hit.

### Secondary structure assessment of designed sequences

To assess the quality of designed sequences, a comparison is held between GAPSSIF and the most recent protein design algorithms, Evolver [16, 17] and EvoDesign [3, 15]. In this analysis, five protein structures are extracted from [15] to evaluate the aforementioned algorithms. For each input structure, EvoDesign announces ten amino acid sequences in ten independent runs. Each run comprises a population of 29000 sequences and 30000 iterations. Also, Evolver is executed on three different types of sequences for each protein of this benchmark as it was mentioned in Background. In addition, GAPSSIF runs ten independent times on the benchmark. For each protein, the size of population is defined based on the number of sub-structures in target structure, and the algorithm is repeated almost 50 iterations.

EvoDesign benefits from a secondary structure predictor in its fitness function with comparable results to PSS-Pred [24] while GAPSSIF uses a development version of PHD [21] called Reprof. In order to have a fair comparison between GAPSSIF and EvoDesign, PSI-Pred [25] is used to have an impartial secondary structure prediction. For this, PSI-Pred, PSS-Pred and Reprof prediction results are compared on five proteins in Table 1. Since GAPSSIF uses Reprof as its fitness function, better performance of GAPSSIF draws on Reprof prediction results would be doubtful. Therefore, secondary structures of designed sequences from GAPSSIF, Evolver and EvoDesign are predicted by PSS-Pred, PSI-Pred and Reprof predictors. Since Evolver does not use any prediction algorithm, the results of PSS-Pred and Reprof are sufficient to compare the accuracy of designed sequences.

**Table 1:** Secondary structure assessment of designed sequences. The predicted secondary structure accuracies of designed sequences by GAPSSIF, EvoDesign and Evolver on five proteins are estimated. PSS-Pred, PSI-Pred and Reprof are used as secondary structure prediction algorithms

PDB ID_Chain	GAPSSIF						EvoDesign						Evolver			
	Reprof%		PSS%		PSI%		Reprof%		PSS%		PSI%		Reprof%		PSS%	
	B	Ave	B	Ave	B	Ave	B	Ave	B	Ave	B	Ave	B	Ave	B	Ave
1ZZK_A	100	100	87.5	80.75	87.5	81.37	82	66.5	83	70.55	83	66.3	88.75	88.33	91.25	87.91
1XTE_A	100	99.74	92.24	86.81	93.96	85.94	69	61.9	85	74.7	81	69.6	88.79	89.22	90.51	89.65
2VOU_A	100	99.93	92.46	89.45	92.46	89.17	78	58.8	84	70.1	84	70.1	90.41	55.58	86.30	83.78
3I4O_A	100	100	91.17	87.20	97.05	89.26	73	59.1	82	60.6	75	64	75	70.58	77.94	68.62
1R26_A	100	100	96.15	91.24	92.30	87.49	84	68.2	95	77.6	95	76.5	91.34	91.02	93.26	92.30

Table 1 illustrates secondary structure assessment of three abovementioned designers, GAPSSIF, EvoDesign and Evolver; such that each designed sequence of each protein is evaluated using three different secondary structure predictors. In other words, the secondary structures of designed sequences obtained by independent executions were predicted by Reprof, PSS-Pred, and PSI-Pred. Thus, columns (*B)* and (*Ave)* in Table 1 respectively indicate maximum and average Q3 among all independent runs. For each protein in Table 1, ten independent runs of EvoDesign and GAPSSIF as well as three different executions of Evolver were used. Comparison in this table firmly corroborates strong performance of the proposed method in PSSIF problem.

Undoubtedly, studying the PSSIF problem is a quantum leap forward in solving protein design, since protein secondary structure represents a primary scaffold of the protein conformation. Successful solution for PSSIF problem by GAPSSIF demonstrates that evolutionary information from naturally occurring proteins can lead IPF problem to an efficient solution. Recent studies have demonstrated that PDB database has reached its completeness [26–28] which means that there are few structures outside PDB.

### Tertiary structure assessment of designed sequences

In this subsection, predicted tertiary structure accuracy of designed sequences is evaluated using I-TASSER [29]. Actually, the ultimate goal of IPF problem is to create proteins with enhanced properties or even novel functions. Inasmuch as, protein structure determines its function, understanding the functional architecture enables us to study this macromolecule more practical.

Thus, five designed sequences by GAPSSIF extracted from [15] are folded by I-TASSER [29] where tertiary structure results are evaluated using *TM-Score* [30, 31], *Assigned SS* and *RMSD* [32]. *TM-Score* represents structural alignment score obtained from TM-align [30] and *Assigned SS* shows the similarity between target and secondary structures taken from DSSP, as well Root Mean Square Deviation, *RMSD,* measures the average distance among atoms of superimposed proteins. In Table 2, *TM-Score* greater than 0.3 indicates that the structural similarity is not random. Moreover, *TM-Score* greater than 0.5 means that 1ZZKA, 1R26A, 1XTE, 3I4O and 2VOU are in the same folding class with the input scaffold which means a relative success in solving IPF problem. The value of mean ± standard deviation (0.77 ± 0.13) for *TM-Score* indicates that all of the predicted tertiary structures of proteins are in the same fold with their respective native structures. In addition, the value of mean ± standard deviation of the *RMSD* is 2.15 ± 0.79. Moreover, the average value of Q3, 79 %, is acceptable because finding appropriate templates highly affects the precision of template-based algorithms such as I-TASSER while the sequence identity of designed sequences is low.

**Table 2:** Tertiary structure assessment of designed sequences. TM-Score, RMSD and Assigned SS measure the predicted tertiary structure accuracy of designed sequences by I-TASSER

	TM-Score		RMSD		Assigned SS Q3 (%)	
	B	Ave	B	Ave	B	Ave
1ZZK_A	0.81	0.58	1.71	2.89	86	78
1XTE_A	0.79	0.54	2.26	3.29	88	71
2VOU_A	0.78	0.40	2.98	3.90	71	66
3I4O_A	0.54	0.43	2.95	3.63	73	66
1R26_A	0.95	0.82	0.88	1.71	81	75
Mean	0.77		2.15		79	

Despite the simplicity of fitness function of GAPSSIF in comparison to EvoDesign and Evolver, the proposed method shows a good performance in designing amino acid sequences. Evolutionary information in both GAPSSIF and EvoDesign can significantly affect designing appropriate sequences for a target scaffold. While, EvoDesign creates a position specific scoring matrix of divergent sequences taken from homolog structures to the target structure, GAPSSIF employs fragments of secondary sub-structures which explicitly participate in building up amino acid sequences. The procedure of assembling amino acid fragments respective to the secondary sub-structures of the target generates protein-like sequences with high diversity. On account of not explicitly considering structural features and the simplicity of the fitness function in proposed method, GAPSSIF shows strong performance in solving PSSIF problem and acceptable results for IPF problem. Furthermore, unfair evaluation of GAPSSIF by homology-based folding algorithms due to low sequence identity negatively affects the evaluation designed sequences. In other words, evolutionary information lends GAPSSIF an ability that improves the designing process in this approach by imposing implicitly tertiary structure constrains which implied by natural data.

### Statistical assessment of designed sequences

In this subsection, two statistical tests are applied to confirm that designed sequences share common characteristics with reference sequences. For this, Pot statistic and Pearson’s chi square tests are employed respectively to measure bunching and inconsistency of the observed amino acid distribution in a designed sequence.

#### Bunching assessment

One of the possible issues in designing artificial sequences is bunching or grouping of a particular amino acid based on the secondary structure state, e.g. β structures are populated by Isoleucine and Valine. Thus, to exclude this possibility, a Pot statistic [16, 33] test is employed to penalize the short-range bunching of particular amino acid in sequence *S* = *s*_1_…*s*_*l*_, as follows:

$$ {E}^{pot}=\frac{1}{l}{\displaystyle {\sum}_{j\in \varSigma }0.5\left(\frac{po{t}_j-\overline{po{t}_j}}{\sigma_j}\right)}- \ln \frac{1}{\sigma_j\sqrt{2}}, $$

where for each amino acid *j*, $\overline{po{t}_j}$ and *σ*_*j*_ assigns to the mean and the corresponding standard deviation calculated for a set of non-redundant PDB native sequences (Brylinski, personal communication). In addition, *pot*_*j*_ is computed as below:

$$ po{t}_j=\frac{po{t}_j^1-po{t}_j^0}{\sigma_j^0}, $$

with

$$ po{t}_j^0=\frac{O_j\left({O}_j-1\right)}{l\left(l-1\right)}\times \frac{r}{1-r}\times \left(l-\frac{1}{1-r}\right), $$

and

$$ po{t}_j^1={\displaystyle {\sum}_{i<k}^{i,k=1,\dots, l}{\chi}_j\left({s}_i\right)\times }{\chi}_j\left({s}_k\right){r}^{\left|i-k\right|}, $$

and

$$ {\sigma}_j^0=\sqrt{\frac{r^2}{1-{r}^2}\times \frac{O_j^2}{l}\times {\left(1-\frac{O_j}{l}\right)}^2}, $$

where *O*_*j*_ shows the frequency of amino acid *j* in sequence *S* as well $r={e}^{-{O}_j/l}$.

For each protein in Additional file 1: Table S1, the *E*^*pot*^ value of designed, reference, bunched and random protein-like sequences are illustrated in Table 3. In fact, the *E*^*pot*^ score of reference sequences are assessed to demonstrate that the bunching of designed sequences is typical of the native protein sequences. Moreover, in order to compare the obtained results, the maximum and minimum bunching are assessed by calculating the *E*^*pot*^ score respectively for bunched and random protein-like sequences. To acquire the maximum bunching, the reference sequences are bunched, e.g. the bunched sequence of DCADCDA is AACCDDD. In addition, the reference sequences are shuffled to generate random protein-like sequence to obtain minimum bunching value.

**Table 3:** Statistical assessment of designed sequences.

PDB ID_Chain	*E* ^*pot*^ (*a*)				*χ* ^2^ *statistic* (*b*)		
	Designed	Reference	Protein-like	Bunched	Designed	Reference	Uniprot-distributed
1ZZK_A	21.90	33.03	24.24	295.21	11.06	37.00	27.08
1XTE_A	41.57	42.78	32.98	429.21	23.28	26.14	15.31
1T3Y_A	46.15	40.46	43.67	652.03	22.07	21.70	8.88
1VQS_A	40.54	44.20	29.42	356.21	39.51	29.21	18.77
1OH0_A	45.88	29.85	34.79	438.79	37.73	25.73	12.06
1A2P_A	30.72	27.89	29.88	309.62	29.50	20.66	9.93
1EW4_A	28.98	35.20	37.23	347.37	17.05	36.39	10.05
1HZT_A	38.60	41.60	37.11	604.35	22.24	19.61	12.79
1IDP_A	46.38	37.74	43.54	634.46	50.10	36.94	17.01
1IUJ_A	38.96	31.30	34.49	402.85	32.30	30.23	21.15
1MG4_A	28.03	38.06	36.36	300.93	38.60	13.89	9.59
1NZ0_A	35.95	51.58	48.39	850.27	30.91	55.66	8.16
1URR_A	30.18	28.02	23.55	192.76	21.58	18.81	18.08
1VH5_A	43.58	33.43	37.03	595.33	27.32	15.73	30.65
1VKK_A	40.07	42.42	38.21	612.99	24.51	22.78	13.62
1WLU_A	38.57	40.23	36.69	613.45	40.15	25.25	13.47
1X6Z_A	32.58	44.50	35.98	642.98	27.93	28.63	29.13
1ZHV_A	38.18	67.38	47.61	722.31	22.84	23.84	17.15
2BWF_A	23.42	16.12	21.48	155.17	27.59	18.33	11.07
2FTR_A	49.27	21.01	36.12	290.87	77.06	34.49	15.43
2GPI_A	21.97	26.50	30.76	327.01	17.54	39.97	16.05
2PV2_A	30.48	32.50	32.56	318.45	24.16	13.83	13.01
3EBT_A	63.24	41.44	52.13	643.93	31.17	29.77	22.43
3EF8_A	56.73	46.87	43.06	704.12	36.44	23.44	19.55
3FEA_A	18.96	26.99	25.63	221.59	21.73	28.18	15.01
1GBS_A	72.44	56.16	46.31	1135.2	44.03	31.58	10.53
1R26_A	34.94	23.04	29.40	248.86	17.64	13.40	16.07
1Y25_A	48.29	53.31	52.55	1134.5	26.18	26.00	19.10
2PTH_A	66.33	77.51	58.86	1671.6	33.55	23.49	20.73
1ABA_A	22.67	18.05	19.76	166.69	15.93	16.75	11.94
1DBW_A	35.58	41.21	36.24	526.48	16.34	22.71	16.45
1I2T_A	34.03	34.00	23.55	182.00	20.22	20.07	10.84
1JF8_A	50.42	41.48	31.59	538.69	28.52	22.40	19.64
1KNG_A	47.69	47.33	39.62	777.74	20.34	24.70	13.16
2CAR_A	78.57	71.70	70.30	1603.3	31.39	26.90	13.87
1MF7_A	64.18	63.88	71.41	1550.0	29.86	20.98	16.92
1SHU_X	66.39	52.18	55.33	1426.0	16.93	19.34	15.08
1BKR_A	25.63	30.22	25.95	308.33	14.10	29.46	19.09
2GMY_A	37.04	34.64	35.70	604.25	29.84	16.53	8.61
1OAI_A	21.36	14.58	14.37	76.785	15.64	23.19	12.30
1UTG_A	23.59	22.73	20.83	142.38	33.41	18.75	16.26
1TQG_A	39.34	35.46	44.32	372.97	25.16	22.52	29.04
1TUK_A	15.96	21.39	25.78	190.00	8.20	73.94	26.47
1ZKE_A	52.57	26.50	39.81	282.41	56.29	24.02	16.89
2J5Y_A	19.41	18.76	23.69	156.59	23.08	19.26	22.90
2P5K_A	15.82	20.15	15.64	87.718	12.72	13.78	31.03
1GUT_A	20.80	34.68	33.24	276.11	24.02	18.78	11.73
2O1Q_A	42.20	35.61	42.15	634.86	33.39	35.21	14.52
3I4O_A	16.56	17.85	21.53	151.22	20.91	18.08	22.34
1EAQ_A	35.55	36.74	38.15	525.38	53.59	18.46	17.31
1JB3_A	35.90	38.39	33.32	444.65	25.70	21.64	14.12
1KMT_A	40.62	50.03	36.15	598.09	33.67	15.22	24.35
1KQ1_A	14.84	13.58	18.51	71.494	21.90	15.49	22.93
1NXM_A	61.59	60.02	60.89	1582.6	42.49	29.20	22.84
1O7I_A	35.83	38.47	43.29	571.87	20.76	21.11	19.77
1OK0_A	21.76	20.51	23.92	151.96	36.62	29.67	22.49
1QHQ_A	41.56	66.12	48.61	1052.2	19.43	59.80	15.67
1R6J_A	13.76	22.35	21.93	234.14	7.06	23.50	27.16
1UCS_A	12.26	20.15	19.60	150.73	21.99	29.24	16.83
2C9Q_A	34.82	31.49	33.78	416.12	22.62	33.69	16.02
2F01_A	33.91	32.49	41.94	632.93	44.30	69.50	17.83
2J2J_A	56.97	53.80	54.79	1232.2	29.27	44.44	14.14
2VMH_A	52.37	48.25	65.53	1022.4	48.85	31.09	23.27
3VUB_A	44.39	27.49	24.28	283.47	31.45	19.60	18.99
1M9Z_A	24.13	32.38	39.90	431.24	34.22	100.74	19.58
2J8B_A	22.71	21.00	24.56	264.71	28.96	105.67	14.13
2VOU_A	36.64	45.43	54.36	937.67	35.35	28.20	22.98
1V5I_B	28.93	20.44	28.45	145.74	31.51	11.70	15.60
2WLV_A	40.35	36.52	41.68	586.89	52.72	31.57	10.40
1F46_A	58.98	43.65	38.99	541.85	36.50	14.21	17.10
1VZI_A	33.33	37.45	42.36	578.66	63.84	39.32	18.86
2ANX_A	49.41	53.04	52.31	744.32	20.30	10.30	14.17
2CMP_A	25.96	25.58	31.02	154.14	19.60	18.92	10.19
2CVI_A	27.21	47.82	37.24	298.96	39.62	39.01	23.82
2D3D_A	27.45	28.78	35.66	306.37	18.22	22.52	15.46
2ERB_A	36.41	36.63	36.23	504.87	17.94	52.38	16.72
2O9S_A	16.01	13.43	15.44	100.61	59.57	14.03	9.57
2PR7_A	40.92	48.59	39.60	857.73	28.04	30.25	24.34
2QCP_X	19.48	19.43	22.94	150.64	25.94	18.24	15.44
2V1Q_A	14.77	19.23	19.72	98.525	20.10	18.11	16.45
2VPB_A	23.28	17.56	22.49	121.38	16.62	65.12	9.10
2VZC_A	40.47	40.82	51.30	660.83	16.20	33.51	20.36
2ZXY_A	31.88	25.72	31.14	312.36	24.89	22.00	21.61
3CTG_A	32.67	31.39	40.23	407.36	27.96	11.53	16.02
3E9T_A	29.23	38.88	39.61	488.97	21.08	28.89	21.48
3FIL_A	18.22	24.39	24.34	126.78	21.55	14.81	17.17
3G21_A	25.86	19.68	18.65	156.69	30.02	17.41	17.60
3G36_A	13.81	14.90	11.31	108.60	21.10	11.57	7.88
3IV4_A	35.41	25.83	31.88	388.37	31.85	26.41	9.82
Mean	35.64	35.30	35.58	498.34	28.71	28.16	17.08
Standard deviation	14.56	14.00	12.58	374.65	12.36	16.88	5.43
Quartile 1	23.59	24.39	24.56	221.59	20.76	18.75	13.47
Median	35.41	34.64	35.98	407.36	26.18	23.49	16.45
Quartile 3	42.20	42.78	42.15	634.46	33.55	30.25	20.36

*(a)* Pot statistic test penalizes short-range bunching of amino acids. The *E* ^*pot*^ value of reference and protein-like sequences give the minimal bunching. On the other hand, the maximal bunching is obtained from bunched sequences. The *E* ^*pot*^ values of designed sequences confirm that their bunching is typical of the native sequences. *(b)* Chi-square test is applied to determine if there is any significant difference between two sets of categorical data. The *χ* ^2^ values indicate that the distribution of designed sequences versus Uniprot database is as significant as reference sequences

Finally, mean, standard deviation, median, quartile 1 and quartile 3 of Table 3 indicate that the amino acid bunching of designed sequences is typical of the reference and random protein-like sequences as well much lower than the bunched sequences.

#### Pearson’s chi-square assessment

Pearson’s chi-square test [34] is applied to sets of categorical data to determine if there is any significant difference between the background (Uniprot [35]) and observed distributions of amino acids in a protein sequence. For each protein *i* with length *l*, we define a random *l*-length sequence according to the amino acid distribution in Uniprot. In the following, chi-square test is calculated on designed, reference and uniprot-distributed sequences versus background:

$$ {\chi}_j^2={\displaystyle {\sum}_{j\in \varSigma}\frac{O_j-{E}_j}{E_j}}, $$

where *O*_*j*_ and *E*_*j*_ are the frequency of amino acid *j* in a protein sequence and Uniprot database, respectively. Table 3 illustrates the obtained chi-square for each designed, reference and uniprot-distributed sequences of a protein. The mean, standard deviation, median, quartile 1 and quartile 3 indicate that the distribution of the designed sequences versus background is as significant as the reference sequences.

## Conclusion

GAPSSIF algorithm performs successful design for its input secondary structure scaffold. Interestingly, the acceptable results for 3D structure in lack of crucial tertiary structure features arise from the effect of evolutionary information. On the other hand, taking into account extra important features such as solvent accessibility and torsion angles, can significantly enhance tertiary structure results.

Using the evolutionary information from proteins with known structures significantly improves the quality of designed sequences. In fact, IPF problem would be solved by applying this information for both 2D and 3D structures. Evidently, in order to have better results in 3D, some effective features such as solvent accessibility and torsion angles should be considered. Therefore, the simple fitness function of GAPSSIF would be improved by a multi-featured one to search through the sequence space more precisely.

## Additional file

Additional file 1: Table S1. GAPSSIF evaluation on a non-redundant dataset. (DOCX 39 kb)

## Abbreviations

AFR: Amino Acid Fragment Repository
AFRM: Amino Acid Fragment Repository Mutation
Assigned SS: Assigned secondary structure
C: Coil
DSSP: Define secondary structure of proteins
E: Beta sheet
GAPSSIF (the name of proposed algorithm): Genetic Algorithm for Protein Secondary Structure Inverse Folding
H: Alpha helix
HMR: Hit Map Repository
HMRM: Hit Map Repository Mutation
IPF: Inverse Protein Folding
PDB: Protein Data Bank
PSP: Protein Structure Prediction
PSSIF: Protein Secondary Structure Inverse Folding
RMSD: Root Mean Square Deviation
SCOP: Structural Classification of Proteins
