# Supplementary material for: Evaluating the accuracy of protein design using native secondary sub-structures
Source: BMC Bioinformatics. 2016 Sep 5;17(1):353. doi: 10.1186/s12859-016-1199-y (PMC5011913; doi:10.1186/s12859-016-1199-y)
Supplement: Additional file 1: Table S1. — GAPSSIF evaluation on a non-redundant dataset. (DOCX 39 kb) [file 12859_2016_1199_MOESM1_ESM.docx]

| PDB ID_Chain | Len | SCOP | PSD-Q3%  $(a)$ | SOV%  $(b)$ | Itr $(c)$ | Enrich time $(d)$  **Seconds** | Search time  $(e)$ | Total time +1 $(f)$ | NDC  $(g)$ | Seq  ID%  $(h)$ | Frag ID $(i)$ | AA comp Var $(j)$ | Num of hits/10 $(k)$ | Ave Q3 $(l)$ | Non-hit  Ave Q3 $(m)$ | PSR-Q3%  $(n)$ |
| --- | --- | --- | --- | --- | --- | --- | --- | --- | --- | --- | --- | --- | --- | --- | --- | --- |
| 1ZZK_A | 80 | α + β | 100 | 100 | 1 | 70 | 9 | 80 | 3.39 | 6 | 0/11 | 0.001089 | 10 | 100 | 0 | 75.0 |
| 1XTE_A | 116 | α + β | 100 | 100 | 7 | 146 | 46 | 193 | 4.11 | 3 | 0/14 | 0.000120 | 8 | 99.74 | 98.70 | 66.4 |
| 1T3Y_A | 131 | α + β | 100 | 100 | 22 | 273 | 243 | 517 | 3.51 | 9 | 0/25 | 0.000674 | 6 | 99.61 | 99.04 | 55.7 |
| 1VQS_A^#^ | 104 | α + β | 100 | 100 | 8 | 155 | 45 | 201 | 2.77 | 7 | 1/19 | 0.000157 | 9 | 99.88 | 99.09 | 49.1 |
| 1OH0_A | 125 | α + β | 100 | 100 | 19 | 211 | 181 | 393 | 3.39 | 8 | 0/22 | 0.002116 | 3 | 99.20 | 98.85 | 52.8 |
| 1A2P_A | 108 | α + β | 100 | 100 | 13 | 192 | 86 | 279 | 3.29 | 6 | 0/21 | 0.000439 | 9 | 99.90 | 99.07 | 51.9 |
| 1EW4_A | 106 | α + β | 100 | 100 | 1 | 138 | 14 | 153 | 3.05 | 10 | 0/18 | 0.000881 | 10 | 100 | 0 | 62.3 |
| 1HZT_A | 153 | α + β | 100 | 100 | 11 | 517 | 119 | 637 | 3.32 | 13 | 0/31 | 0.000193 | 4 | 99.60 | 99.34 | 45.8 |
| 1IDP_A | 147 | α + β | 100 | 100 | 6 | 224 | 45 | 270 | 4.35 | 7 | 0/22 | 0.000100 | 7 | 99.79 | 99.59 | 42.9 |
| 1IUJ_A | 102 | α + β | 100 | 100 | 4 | 151 | 26 | 178 | 4.07 | 5 | 0/15 | 0.000417 | 9 | 99.90 | 99.01 | 52.0 |
| 1MG4_A | 101 | α + β | 100 | 100 | 4 | 123 | 22 | 146 | 3.18 | 9 | 0/15 | 0.000967 | 10 | 100 | 0 | 52.5 |
| 1NZ0_A | 109 | α + β | 100 | 100 | 4 | 158 | 24 | 183 | 4.49 | 8 | 0/19 | 0.001138 | 9 | 99.72 | 97.24 | 51.4 |
| 1URR_A^#^ | 97 | α + β | 100 | 100 | 1 | 122 | 13 | 136 | 3.19 | 8 | 1/17 | 0.000014 | 10 | 100 | 0 | 47.4 |
| 1VH5_A | 138 | α + β | 100 | 100 | 4 | 238 | 33 | 272 | 3.02 | 5 | 0/23 | 0.000471 | 10 | 100 | 0 | 50.0 |
| 1VKK_A | 137 | α + β | 100 | 100 | 8 | 323 | 61 | 385 | 4.31 | 5 | 0/27 | 0.001360 | 10 | 100 | 0 | 51.8 |
| 1WLU_A | 117 | α + β | 100 | 100 | 5 | 210 | 34 | 245 | 4.27 | 7 | 0/19 | 0.002820 | 8 | 99.74 | 98.71 | 69.2 |
| 1X6Z_A | 120 | α + β | 100 | 100 | 5 | 267 | 35 | 303 | 4.32 | 10 | 0/25 | 0.000266 | 7 | 99.66 | 98.88 | 40.8 |
| 1ZHV_A | 134 | α + β | 100 | 100 | 4 | 199 | 31 | 231 | 3.33 | 9 | 0/21 | 0.000083 | 10 | 100 | 0 | 55.2 |
| 2BWF_A | 77 | α + β | 100 | 100 | 11 | 143 | 61 | 205 | 2.68 | 12 | 0/16 | 0.001083 | 8 | 99.74 | 98.70 | 62.3 |
| 2FTR_A | 103 | α + β | 100 | 100 | 3 | 173 | 20 | 194 | 4.37 | 2 | 0/18 | 0.006071 | 9 | 99.90 | 99.02 | 54.4 |
| 2GPI_A | 91 | α + β | 100 | 100 | 4 | 107 | 20 | 128 | 3.00 | 3 | 0/16 | 0.000358 | 10 | 100 | 0 | 51.6 |
| 2PV2_A | 103 | α + β | 100 | 100 | 9 | 155 | 50 | 206 | 2.93 | 7 | 0/18 | 0.000089 | 9 | 99.90 | 99.02 | 57.3 |
| 3EBT_A | 132 | α + β | 100 | 100 | 5 | 214 | 35 | 250 | 3.89 | 11 | 0/22 | 0.001083 | 10 | 100 | 0 | 54.5 |
| 3EF8_A | 149 | α + β | 100 | 100 | 7 | 226 | 55 | 282 | 4.14 | 7 | 0/22 | 0.000250 | 10 | 100 | 0 | 53.0 |
| 3FEA_A | 83 | α + β | 100 | 100 | 11 | 159 | 61 | 221 | 3.07 | 13 | 0/20 | 0.000693 | 8 | 99.63 | 98.19 | 59.0 |
| 1GBS_A | 185 | α + β | 100 | 100 | 6 | 468 | 73 | 542 | 5.39 | 7 | 0/31 | 0.000002 | 10 | 100 | 0 | 35.1 |
| 1R26_A | 104 | α/β | 100 | 100 | 5 | 193 | 30 | 224 | 2.37 | 10 | 0/21 | 0.002280 | 10 | 100 | 0 | 61.5 |
| 1Y25_A* | 165 | α/β | 100 | 100 | 8 | 725 | 106 | 832 | 5.84 | 4 | 0/32 | 0.001083 | 7 | 99.75 | 99.19 | 43.6 |
| 2PTH_A | 193 | α/β | 100 | 100 | 7 | 484 | 95 | 580 | 4.62 | 10 | 0/31 | 0.000174 | 10 | 100 | 0 | 59.6 |
| 1ABA_A | 87 | α/β | 100 | 100 | 2 | 146 | 15 | 162 | 2.66 | 14 | 0/15 | 0.000144 | 10 | 100 | 0 | 56.3 |
| 1DBW_A | 123 | α/β | 100 | 100 | 2 | 193 | 21 | 215 | 3.24 | 13 | 0/21 | 0.003115 | 10 | 100 | 0 | 66.7 |
| 1I2T_A | 61 | α/β | 100 | 100 | 3 | 34 | 10 | 45 | 1.58 | 8 | 0/9 | 0.000635 | 10 | 100 | 0 | 83.6 |
| 1JF8_A | 130 | α/β | 100 | 100 | 1 | 196 | 19 | 216 | 3.41 | 8 | 0/19 | 0.000017 | 10 | 100 | 0 | 57.7 |
| 1KNG_A | 144 | α/β | 100 | 100 | 8 | 480 | 71 | 552 | 3.30 | 7 | 0/33 | 0.002820 | 6 | 99.72 | 99.30 | 50.7 |
| 2CAR_A | 196 | α/β | 100 | 100 | 9 | 489 | 128 | 618 | 4.63 | 9 | 0/29 | 0.000069 | 9 | 99.89 | 99.89 | 55.1 |
| 1MF7_A | 194 | α/β | 100 | 100 | 11 | 660 | 169 | 830 | 3.86 | 5 | 0/38 | 0.000081 | 9 | 99.94 | 99.48 | 52.1 |
| 1SHU_X | 181 | α/β | 100 | 100 | 4 | 498 | 61 | 560 | 4.29 | 9 | 0/28 | 0.000191 | 10 | 100 | 0 | 67.4 |
| 1BKR_A | 108 | α | 100 | 100 | 12 | 124 | 78 | 203 | 3.21 | 7 | 0/17 | 0.000439 | 7 | 99.72 | 99.07 | 52.8 |
| 2GMY_A | 147 | α | 100 | 100 | 5 | 170 | 36 | 207 | 3.18 | 7 | 0/19 | 0.000558 | 9 | 99.93 | 99.31 | 63.3 |
| 1OAI_A | 59 | α | 100 | 100 | 1 | 34 | 6 | 41 | 2.66 | 5 | 0/9 | 0.001685 | 10 | 100 | 0 | 78.0 |
| 1UTG_A | 70 | α | 100 | 100 | 8 | 50 | 28 | 79 | 2.97 | 8 | 0/11 | 0.000229 | 5 | 99 | 98.33 | 71.4 |
| 1TQG_A | 105 | α | 100 | 100 | 1 | 39 | 8 | 48 | 3.54 | 6 | 0/9 | 0.000107 | 10 | 100 | 0 | 80.0 |
| 1TUK_A | 67 | α | 100 | 100 | 2 | 34 | 8 | 43 | 3.13 | 7 | 0/9 | 0.000792 | 10 | 100 | 0 | 38.8 |
| 1ZKE_A | 81 | α | 100 | 100 | 1 | 23 | 5 | 29 | 3.46 | 7 | 0/7 | 0.000430 | 10 | 100 | 0 | 72.8 |
| 2J5Y_A | 61 | α | 100 | 100 | 1 | 21 | 5 | 27 | 2.83 | 11 | 0/7 | 0.000574 | 10 | 100 | 0 | 86.9 |
| 2P5K_A | 63 | α | 100 | 100 | 1 | 50 | 7 | 58 | 2.35 | 3 | 0/11 | 0.000456 | 10 | 100 | 0 | 36.5 |
| 1GUT_A | 67 | β | 100 | 100 | 19 | 78 | 119 | 198 | 3.22 | 7 | 0/14 | 0.011274 | 3 | 98.05 | 97.57 | 59.7 |
| 2O1Q_A | 144 | β | 100 | 100 | 19 | 506 | 237 | 744 | 5.44 | 7 | 0/34 | 0.000020 | 5 | 99.44 | 98.88 | 41.0 |
| 3I4O_A | 68 | β | 100 | 100 | 1 | 68 | 8 | 77 | 2.61 | 8 | 0/13 | 0.003557 | 10 | 100 | 0 | 63.2 |
| 1EAQ_A | 124 | β | 100 | 100 | 3 | 355 | 31 | 387 | 4.46 | 7 | 0/29 | 0.003014 | 10 | 100 | 0 | 36.3 |
| 1JB3_A | 127 | β | 100 | 100 | 6 | 230 | 40 | 271 | 3.32 | 3 | 0/23 | 0.002728 | 10 | 100 | 0 | 52.0 |
| 1KMT_A | 138 | β | 100 | 100 | 1 | 278 | 23 | 302 | 3.30 | 5 | 0/23 | 0.000838 | 10 | 100 | 0 | 62.3 |
| 1KQ1_A | 60 | β | 100 | 100 | 4 | 67 | 14 | 82 | 2.12 | 8 | 0/13 | 0.001764 | 10 | 100 | 0 | 61.7 |
| 1NXM_A | 194 | β | 99.4 | 99.74 | 7 | 942 | 124 | 1067 | 4.04 | 10 | 0/38 | 0.000598 | 0 | 99.22 | 99.22 | 32.0 |
| 1O7I_A | 115 | β | 100 | 100 | 5 | 247 | 35 | 283 | 4.05 | 7 | 0/20 | 0.001417 | 10 | 100 | 0 | 60.0 |
| 1OK0_A | 74 | β | 100 | 100 | 1 | 114 | 11 | 126 | 2.21 | 9 | 0/17 | 0.002510 | 10 | 100 | 0 | 60.8 |
| 1QHQ_A | 139 | β | 100 | 100 | 2 | 419 | 34 | 454 | 5.18 | 7 | 0/21 | 0.002491 | 10 | 100 | 0 | 47.5 |
| 1R6J_A | 82 | β | 100 | 100 | 3 | 119 | 15 | 135 | 2.68 | 9 | 0/17 | 0.000008 | 10 | 100 | 0 | 50.0 |
| 1UCS_A | 64 | β | 100 | 100 | 8 | 185 | 52 | 238 | 2.59 | 3 | 0/12 | 0.004489 | 8 | 99.68 | 98.43 | 34.4 |
| 2C9Q_A | 102 | β | 100 | 100 | 1 | 152 | 15 | 168 | 4.40 | 6 | 0/19 | 0.001603 | 10 | 100 | 0 | 57.8 |
| 2F01_A | 121 | β | 100 | 100 | 1 | 163 | 16 | 180 | 5.87 | 10 | 0/19 | 0.000070 | 10 | 100 | 0 | 66.1 |
| 2J2J_A | 182 | β | 100 | 100 | 20 | 763 | 393 | 1157 | 5.12 | 3 | 0/34 | 0.000894 | 3 | 98.84 | 98.35 | 52.7 |
| 2VMH_A | 147 | β | 100 | 100 | 14 | 458 | 147 | 606 | 6.47 | 8 | 0/32 | 0.000926 | 4 | 99.18 | 98.63 | 47.6 |
| 3VUB_A | 101 | β | 100 | 100 | 38 | 151 | 518 | 670 | 3.52 | 2 | 0/19 | 0.002591 | 1 | 97.82 | 97.57 | 47.5 |
| 1M9Z_A | 105 | small | 100 | 100 | 11 | 280 | 73 | 354 | 3.69 | 9 | 0/26 | 0.000107 | 6 | 91.23 | 78.09 | 51.4 |
| 2J8B_A | 78 | small | 100 | 100 | 10 | 117 | 46 | 164 | 3.62 | 8 | 0/17 | 0.004928 | 8 | 99.87 | 98.71 | 34.6 |
| 2VOU_A | 146 | - | 100 | 100 | 4 | 405 | 50 | 456 | 5.18 | 15 | 0/28 | 0.002661 | 9 | 99.93 | 99.31 | 68.5 |
| 1V5I_B* | 76 | - | 100 | 100 | 2 | 114 | 13 | 128 | 3.11 | 7 | 0/17 | 0.000028 | 10 | 100 | 0 | 50.0 |
| 2WLV_A* | 144 | - | 100 | 100 | 2 | 170 | 22 | 193 | 4.29 | 9 | 0/19 | 0.000641 | 10 | 100 | 0 | 55.6 |
| 1F46_A | 139 | - | 100 | 100 | 33 | 299 | 552 | 852 | 4.23 | 14 | 0/26 | 0.000045 | 1 | 98.41 | 98.24 | 66.2 |
| 1VZI_A | 125 | - | 100 | 100 | 12 | 409 | 95 | 505 | 4.57 | 8 | 0/31 | 0.002916 | 6 | 99.68 | 99.2 | 31.2 |
| 2ANX_A | 146 | - | 100 | 100 | 3 | 481 | 43 | 525 | 3.39 | 6 | 0/21 | 0.000101 | 9 | 99.93 | 99.31 | 54.8 |
| 2CMP_A^#^ | 56 | - | 100 | 100 | 1 | 34 | 6 | 41 | 3.21 | 1 | 1/9 | 0.000180 | 10 | 100 | 0 | 80.4 |
| 2CVI_A | 83 | - | 100 | 100 | 7 | 117 | 35 | 153 | 2.77 | 6 | 0/15 | 0.000204 | 9 | 98.79 | 98.79 | 45.8 |
| 2D3D_A | 83 | - | 100 | 100 | 18 | 118 | 126 | 245 | 3.63 | 9 | 0/17 | 0.001149 | 6 | 99.51 | 98.79 | 50.6 |
| 2ERB_A*^#^ | 123 | - | 100 | 100 | 1 | 131 | 14 | 146 | 4.31 | 6 | 1/17 | 0.000230 | 10 | 100 | 0 | 61.8 |
| 2O9S_A | 67 | - | 100 | 100 | 8 | 112 | 46 | 159 | 2.74 | 5 | 0/11 | 0.000002 | 10 | 100 | 0 | 52.2 |
| 2PR7_A | 137 | - | 100 | 100 | 3 | 240 | 28 | 269 | 4.38 | 13 | 0/23 | 0.004367 | 10 | 100 | 0 | 58.4 |
| 2QCP_X | 80 | - | 100 | 100 | 4 | 133 | 20 | 154 | 2.61 | 8 | 0/15 | 0.000870 | 8 | 99.75 | 98.75 | 57.5 |
| 2V1Q_A | 60 | - | 100 | 100 | 13 | 111 | 87 | 199 | 2.37 | 8 | 0/11 | 0.000641 | 3 | 98.66 | 98.09 | 41.7 |
| 2VPB_A^#^ | 57 | - | 100 | 100 | 23 | 98 | 149 | 248 | 2.57 | 12 | 1/16 | 0.000524 | 1 | 95.26 | 94.73 | 36.8 |
| 2VZC_A | 127 | - | 100 | 100 | 1 | 80 | 11 | 92 | 4.07 | 9 | 0/13 | 0.001331 | 10 | 100 | 0 | 55.9 |
| 2ZXY_A | 86 | - | 100 | 100 | 3 | 71 | 15 | 87 | 3.08 | 5 | 0/13 | 0.000534 | 10 | 100 | 0 | 51.2 |
| 3CTG_A | 108 | - | 100 | 100 | 10 | 186 | 59 | 246 | 3.78 | 10 | 0/21 | 0.001196 | 9 | 99.07 | 99.07 | 63.9 |
| 3E9T_A | 112 | - | 100 | 100 | 1 | 226 | 31 | 258 | 2.92 | 2 | 0/23 | 0.002415 | 10 | 100 | 0 | 64.3 |
| 3FIL_A* | 56 | - | 100 | 100 | 5 | 57 | 15 | 73 | 2.72 | 10 | 0/12 | 0.000978 | 10 | 100 | 0 | 75.0 |
| 3G21_A | 77 | - | 100 | 100 | 3 | 51 | 12 | 64 | 3.39 | 9 | 0/11 | 0.000036 | 10 | 100 | 0 | 49.4 |
| 3G36_A | 52 | - | 100 | 100 | 1 | 33 | 6 | 40 | 2.70 | 3 | 0/9 | 0.000358 | 9 | 98.07 | 98.07 | 65.4 |
| 3IV4_A | 112 | - | 100 | 100 | 19 | 193 | 157 | 351 | 2.57 | 11 | 0/21 | 0.000499 | 6 | 99.28 | 98.21 | 60.7 |

**Table 1*.* GAPSSIF evaluation on a non-redundant dataset.**  PDB ID_Chains of 89 non-redundant proteins with different lengths and folding classes is used to evaluate the proposed method (GAPSSIF). The length of selected proteins varies from 52 to 196 amino acids, as it is shown in the second column. The third column shows the folding class according to SCOP, e.g. 9 alpha (α), 18 beta (β), 26 alpha+beta (α + β), 11 alpha/beta (α/β) and 2 small proteins. In addition, (-) assigns to proteins with no SCOP family. Other columns of the table are describes as bellow:

***(a):*** is the fraction of correctly designed residues in the predicted secondary structure; ***(b):*** illustrates the segment overlap score; ***(c):*** represents the termination iterations of proposed algorithm; ***(d)* and *(e):*** respectively demonstrate enrichment and searching time for each protein (Note: all times are in seconds); ***(f):*** shows the total time of the algorithm; ***(g):*** is the amino acid composition difference between designed and its corresponding reference sequence; ***(h)* and *(i):*** respectively indicate sequence and corresponding fragments identity in the reference and designed sequences; ***(j):*** represents the value of variance between amino acids of designed and reference sequences; ***(k):*** shows the number of designed sequences whose secondary structures are identical to the target structure among ten independent executions for each protein; ***(l):*** is the average accuracy of all ten designed sequences; ***(m):*** is the average accuracy of non-hit designed sequences out of ten executions; **(n):** shows the fraction of residues which their predicted secondary structures, using Reprof, are identical to the native structure. In the first column signs “#”, “*” represent respectively proteins with non-zero fragment identity and omitted proteins from the repository (AFR).
